# Recolonizing carnivores: Is cougar predation behaviorally mediated by bears?

**DOI:** 10.1002/ece3.7424

**Published:** 2021-03-21

**Authors:** Kristin N. Engebretsen, Jon P. Beckmann, Carl W. Lackey, Alyson Andreasen, Cody Schroeder, Pat Jackson, Julie K. Young

**Affiliations:** ^1^ Deparment of Wildland Resources Utah State University Logan UT USA; ^2^ Wildlife Conservation Society Bozeman MT USA; ^3^ Game Division Nevada Department of Wildlife Reno NV USA; ^4^ Natural Resources and Environmental Sciences University of Nevada‐Reno Reno NV USA; ^5^ USDA‐NWRC‐Predator Research Facility Millville UT USA; ^6^ Present address: Kansas Department of Wildlife, Parks, and Tourism Pratt KS USA

**Keywords:** Black bear, feral horses, intraguild competition, kleptoparasitism, mule deer, *Puma concolor*, scavenging, *Ursus americanus*

## Abstract

Conservation and management efforts have resulted in population increases and range expansions for some apex predators, potentially changing trophic cascades and foraging behavior. Changes in sympatric carnivore and dominant scavenger populations provide opportunities to assess how carnivores affect one another. Cougars (*Puma concolor*) were the apex predator in the Great Basin of Nevada, USA, for over 80 years. Black bears (*Ursus americanus*) have recently recolonized the area and are known to heavily scavenge on cougar kills. To evaluate the impacts of sympatric, recolonizing bears on cougar foraging behavior in the Great Basin, we investigated kill sites of 31 cougars between 2009 and 2017 across a range of bear densities. We modeled the variation in feeding bout duration (number of nights spent feeding on a prey item) and the proportion of primary prey, mule deer (*Odocoileus hemionus*), in cougar diets using mixed‐effects models. We found that feeding bout duration was driven primarily by the size of the prey item being consumed, local bear density, and the presence of dependent kittens. The proportion of mule deer in cougar diet across all study areas declined over time, was lower for male cougars, increased with the presence of dependent kittens, and increased with higher bear densities. In sites with feral horses (*Equus ferus)*, a novel large prey, cougar consumption of feral horses increased over time. Our results suggest that higher bear densities over time may reduce cougar feeding bout durations and influence the prey selection trade‐off for cougars when alternative, but more dangerous, large prey are available. Shifts in foraging behavior in multicarnivore systems can have cascading effects on prey selection. This study highlights the importance of measuring the impacts of sympatric apex predators and dominant scavengers on a shared resource base, providing a foundation for monitoring dynamic multipredator/scavenger systems.

## Graphical abstract

Cougars have recently experienced the recolonization of black bears in the Great Basin, Nevada, USA, and are known to scavenge on cougar kills. We investigated the variation in feeding bout duration and the proportion of mule deer in cougar diets between 2009 and 2017 across a range of bear densities. We found that feeding bout duration was driven primarily by the size of the prey item being consumed, local bear density, and the presence of dependent kittens, highlighting the importance of measuring the combined impacts of sympatric apex predators on shared resources and provides a foundation for monitoring dynamic multipredator systems.

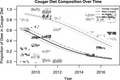

## 1 INTRODUCTION

Populations of many apex predators have declined globally from causes such as habitat fragmentation, prey loss, overexploitation, and human persecution (Ripple et al., 2014; Younger et al., 2016). However, this trend has recently been reversed in some areas due to intensive conservation and management efforts. Large carnivores and facultative scavengers are recovering or have been reintroduced in many systems around the world, including wolves (*Canis lupus*) and lynx (*Lynx lynx*) in Europe (Kuijper et al., 2019), orca (*Orcinus orca*) in the Canadian Arctic (Lefort et al., 2020), and black bears (*Ursus americanus*) in the Great Basin Desert, USA (Beckmann & Lackey, 2018). Changes in population dynamics of large carnivores have been shown to impact trophic cascades (e.g., Ripple et al., 2014), but less is known about how recoveries or reintroductions of a predator or scavenger species impact an existing predator population (Bartnick et al., 2013; Harihar et al., 2011). This is particularly true when one competitor in a system was absent or found historically at very low densities but now has increased due to the absence of the other competitor. Understanding the competitive dynamics of sympatric intraguild predators and scavengers feeding on common resources is critical, as predation and competition are two of the most important ecological processes that structure natural communities (Chase et al., 2002; Clark et al., 2014; Elbroch et al., 2015; Krofel et al., 2012).

Large carnivores can alter the behavior and survival of both their competitors and their prey in a variety of direct and indirect ways (Dröge et al., 2017; Lima & Bednekoff, 1999; Messier, 1994; Winnie & Creel, 2017). However, large carnivore species are not ecologically equivalent in their ability to locate and subdue prey, and therefore, they impact prey communities differently (Elbroch & Kusler, 2018). Allospecific carnivores may interact through exploitative competition, where resources are depleted more efficiently by the superior competitor, or through interference competition, where individuals directly compete to block others from acquiring a resource (Elbroch & Kusler, 2018). Interference interactions between predator and dominant scavenger species, such as kleptoparasitism or food stealing, can negatively impact the subordinate predator through the loss of resources and increased energy expenditure to obtain prey (Orning, 2019; Wilson & Wolkovich, 2011). This competition may alter local prey abundance and distribution, necessitating a better understanding of competitive interactions between predators and facultative scavengers and the subsequent impacts on shared prey species (Knopff et al., 2010; Sinclair, 1985; Wilson & Wolkovich, 2011; Winnie & Creel, 2017).

Cougars (*Puma concolor*) have an expansive range in the Western Hemisphere. In the American West, they often prey on mule deer (*Odocoileus hemionus*), thus emulating a simple “single‐predator, single‐prey” system. However, mule deer fluctuate in abundance across their range (Bleich & Taylor, 1998; Robinson et al., 2002; Wielgus, 2017), and cougars readily consume a variety of secondary ungulate prey, such as elk, caribou, and moose, as well as smaller prey, such as mesocarnivores, birds, and small mammals. Some cougars specialize on alternative prey (e.g., Lowrey et al., 2016). Cougars are solitary hunters and habitually cache their large ungulate kills for feeding bouts over several days. Across their diverse range, cougars are dominant competitors in their interactions with mesopredators, such as coyotes (*Canis latrans*) or ocelots (*Leopardus pardalis*), but they are subordinate competitors when sympatric with large carnivores, such as black bears, wolves, and jaguars (*Panthera onca*; Elbroch & Kusler, 2018, Elbroch et al., 2015).

Historically, both cougars and black bears were thought to have been widely distributed in the Great Basin Desert, although at low densities (Berger & Wehaussen, 1991; Lackey et al., 2013). However, like many large carnivores, black bears were extirpated throughout Nevada by the early 1900s due to landscape‐scale habitat loss, targeted removals, and unmanaged hunting (Beckmann & Lackey, 2018; Lackey et al., 2013). At the same time, a change from a grass‐dominated biome to a sagebrush‐steppe ecosystem created by increased livestock grazing facilitated the irruption of mule deer herds (Berger & Wehausen, 1991; Miller et al., 1994; Strand et al., 2014) and the resulting concomitant increase in the cougar population. This expansive growth of both mule deer and cougar populations occurred as bears were being extirpated, allowing cougars to dominate the predatory landscape in western Nevada for nearly a century. Habitat restoration efforts and a change in management strategies of carnivores throughout the Great Basin have resulted in black bears recolonizing parts of Nevada beginning in the 1980s (Beckmann & Lackey, 2018; Lackey et al., 2013). Bears began expanding across their historic range in the state where they had been absent for almost a century (Lackey et al., 2013; Malaney et al., 2017), with current population estimates suggesting 600–700 bears in western Nevada (NDOW, 2018).

Although cougars will defend their cached prey in interactions where they are the dominant competitor (i.e., against coyotes or other mesocarnivores), cougars are most likely to abandon their kills to other large carnivores or dominant scavengers (Elbroch & Kusler, 2018). Kleptoparasitism by carnivores that also scavenge, such as black bears, can affect the fitness of the losing competitor through negative impacts on their foraging efficiency and individual fitness (Krofel et al., 2012). These negative effects can depress reproductive rates and limit the recruitment of juveniles into the predator population (Orning, 2019). Additionally, kleptoparasitism may drive the predator losing its prey to increase kill rates on primary prey or induce prey switching to secondary prey species, which can either stabilize a food web or create a negative cascading effect, dependent on the other system‐specific factors (Krofel et al., 2012; Wilson & Wolkovich, 2011). Kleptoparasitism of predator kills in different systems has been shown to both increase (Elbroch et al., 2015; Elbroch & Witmer, 2013; Krofel et al., 2012) and decrease (Orning, 2019; Tallian et al., 2017) kill rates by carnivores on their primary prey, highlighting the variability and complexity of these multipredator systems across the globe.

The ongoing recolonization of bears across western Nevada combined with a long‐term dataset detailing that process (e.g., Beckmann & Lackey, 2018) provided an opportunity for a unique natural experiment to assess the behavioral response of a naïve and subordinate competitor, the cougar, to scavenging pressure from a dominant facultative scavenger, the black bear. We utilized seven years of data on cougar predation behavior in combination with the long‐term dataset on recolonizing bear density to determine whether the increasing presence of recolonizing black bears across their historic range in Nevada influenced cougar feeding bout duration and prey composition. We hypothesized that cougars would respond to the growing bear density and kleptoparasitism pressure by spending fewer nights feeding at each prey item. We also hypothesized that cougars experiencing kleptoparasitism would prey more frequently on smaller mammals that they can consume quickly rather than on adult mule deer or horses, thus reducing their risk of losing valuable biomass to scavengers. The goal of our study was to examine predation and competition in populations of sympatric predators and dominant scavengers where the interspecific competition for prey resources is relatively novel.

## 2 MATERIALS AND METHODS

### 2.1 Data collection and processing

**FIGURE 1:**
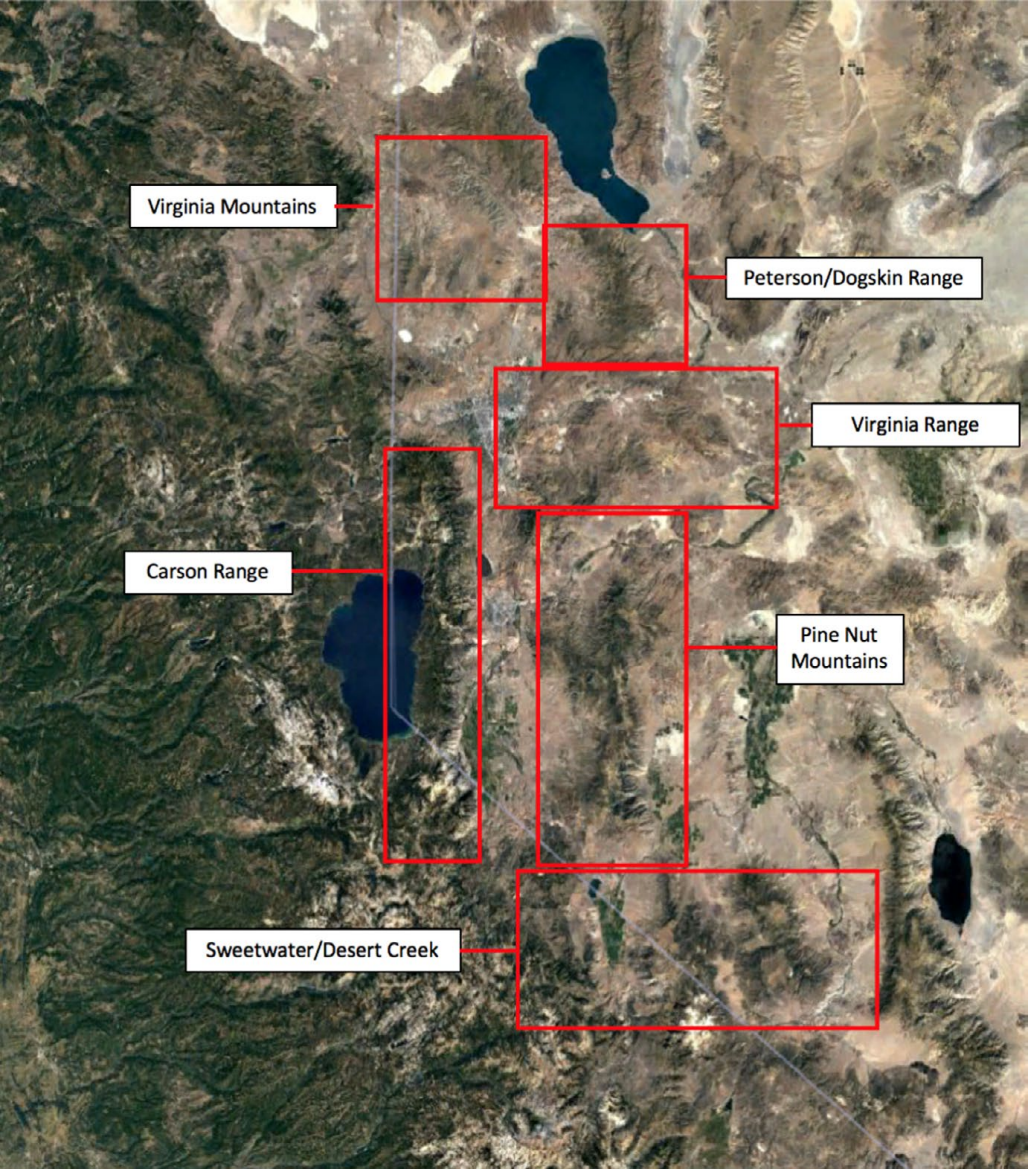
Investigations of cougar kill sites were conducted across several regions of Nevada's Great Basin and Sierra Nevada in the western United States. The Sierra Nevada consists of the Carson Range. The Great Basin contains the other 5 study areas, all of which had feral horses present. The mountain ranges with few to no bears included the Peterson/Dogskin Range, the Virginia Range, and the Virginia Mountains. The ranges with established and growing populations of bears included the Sweetwater/Desert Creek, the Carson Range, and the Pine Nut

We monitored 31 GPS‐collared cougars in Nevada between 2009–2012 and 2015–2017. Cougars were captured, chemically immobilized, and fitted with global positioning system (GPS) collars (Globalstar collars by Vectronic Aerospace GmbH, Berlin, Germany; North Star Science and Technology, King George, Virginia, USA), following approved handling and capture techniques described in Andreasen et al., (2018; State of Nevada scientific collection permit #S33313 and University of Nevada, Reno Animal Care Protocol #A06/07‐28). The GPS collars were programmed to collect geographic coordinates at intervals ranging from 2.5 to 5 hr. The focal study sites were located in the far western edge of the Great Basin and the eastern Sierra Nevada across several mountain ranges (Andreasen et al., 2018; Figure 1). Available prey in the Sierra Nevada consists primarily of mule deer and nonungulate mammals. In the western Great Basin, feral horses (*Equus ferus*) are also present and are a frequent prey item for cougars, in addition to mule deer, bighorn sheep (*Ovis canadensis*), pronghorn (*Antilocapra americana*), livestock, and nonungulate mammals (Andreasen, Stewart, Longland, & Beckmann, *in review*). We conducted kill‐site investigations at clusters of cougar GPS points which were identified using the algorithm developed by Knopff et al. (2009), as likely to contain a cougar kill. We established the criteria for a kill site to be ≥2 GPS points within 200m, including at least one location obtained overnight. We prioritized visiting all clusters with a 25% or greater probability of containing a kill and then searched as many clusters with a probability <25% as the field crew could successfully visit. For each prey item located at each kill‐site location, we identified the species, as well as sex and age where possible. The age of ungulates was determined using tooth eruption and wear. We documented signs of other predators or scavengers at the carcass location.

Each kill‐site investigation record was located within one of six study areas that we delineated based on geography and NDOW wildlife management zones (Figure 1). To evaluate the impact of primary prey availability on cougar predation behavior, we used NDOW estimates of the local density of mule deer for the six study areas (which consist of one or multiple deer management units) in each year (Big Game Status Report, NDOW, 2017). We calculated black bear density for each study area (again consisting of one or multiple NDOW management units) in each year from the long‐term NDOW bear monitoring research (e.g., Beckmann & Berger, 2003a, Beckmann & Lackey, 2008, Lackey et al., 2013, Beckmann & Lackey, 2018; Figure S1). We created a binary variable for horse presence in each study area.

We focused our field data collection on cougar predation behavior at times when cougars most frequently encounter black bears (i.e., bear active season). Thus, we only used kill‐site investigation data for cougar kills made between 1 March and 31 October of each year for these analyses (Beckmann & Berger, 2003a, 2003b). The use of seasonal data differs from another study of cougars in the area with foraging behavior from year‐long data (Andreasen et al., 2021). We used a binary variable to account for the presence of any‐age dependent kittens with an adult female. We also used a second binary variable to account for the presence of kittens older than 3 months, as previous studies indicate that kittens over 3 months significantly contribute to their mother's predation and consumption rates due to the nutritional demands of lactation and kitten consumption of meat (Knopff et al., 2010). These two variables were never included in the same proposed model. We created a binary covariate defined as “Bear Visit” if there was evidence that a bear had found and scavenged at the carcass by the time the kill‐site investigation was conducted (Table 1; Figure S2). We were confident that these identified bear visits involved the bear feeding on the carcass remains in each case by documenting copious amounts of bear scat, tracks, and evidence of bear feeding behavior (such as the skin on limbs peeled back and scattered rumen/remains) at or on the carcass. However, due to the variable time lag between the cluster formation and kill‐site investigation, we were unable to further divide these scavenging events into “active” or “passive” scavenges, based on if the cougar was still actively feeding at the carcass when a bear‐scavenge event occurred. Thus, we classified all bear scavenging behavior as “bear visitation of the kill” to account for both passive and active bear scavenging events.

**TABLE 1:** Percent of cougar kill sites (*N* = number of total prey items) visited by bears between March and October 2009–2012 and 2015–2017, in the three study sites of Sierra Nevada (Carson Range) and Great Basin (Pine Nut and Sweetwater Range), Nevada, USA, with resident bear populations.

Year	Sierra Nevada	Great Basin	
	Carson Range	Pine Nut	Sweetwater
2009	36.36% (*N* = 22)	26.09% (23)	8.33% (12)
2010	47.76% (67)	30.00% (20)	0.00% (2)
2011	36.60% (153)	0.00% (3)	0.00% (2)
2012	(0)	0.00% (3)	12.50% (8)
2015	63.16% (38)	28.57% (28)	33.33% (3)
2016	36.84% (38)	40.00% (20)	0.00% (2)
2017	76.19% (21)	19.44% (36)	9.09% (11)

Similar to our inability to differentiate between active and passive bear scavenging, the time delay between cluster formation and kill investigation in the last several years of the study often precluded field crews from confirming that the prey item in question was indeed a confirmed kill by the cougar and was not a scavenge. To account for this uncertainty, we decided to analyze all kill‐site investigations of prey items that were fed on by cougars, regardless if they were confirmed kills, confirmed scavenges, or unknowns. However, we did remove 11 records from a collared and partially independent subadult which were shared kills with her mother. We did retain prey items that this collared subadult killed or fed upon independently of her mother.

We screened all variables for collinearity (>0.7) using Pearson's correlation. Horse presence was significantly correlated (>0.7) with deer density, bear density, and study site. We removed the horse presence covariate from the possible set of variables for this model set, as it was not biologically critical to the focus of our analysis. Additionally, we can easily separate data with and without horses by the study site. Bear density and deer density were also correlated (>0.7), but because we wanted to test the influence of both bear and deer density on cougar predation behavior, we retained both variables in the possible set to develop our hypothetical models. Bear and deer densities were never used in the same proposed model to avoid multicollinearity.

### 2.2 Feeding bout duration

Individual kills were assigned average sex‐ and age‐specific live weights (Ferguson, 2005; Reid, 2006). We then assigned each prey record to one of 5 weight class categories, using the estimated live weights. The five weight classes were extra‐small, small, medium, large, and extra‐large. The extra‐large prey class (>90 kg/200 lbs) consisted of yearling, subadult, and adult feral horses and domestic cattle. The large class (45–90 kg/100–200 lbs) consisted of adult and subadult mule deer, bighorn sheep, domestic sheep, pronghorn, and feral goats. The medium‐weight class (22.5–45 kg/50–100 lbs) almost exclusively included mule deer fawns and yearlings, from 6 months old to 2 years old. The small prey class (7–22.5 kg/15–50 lbs) contained neonate mule deer from 2 to 6 months old and a variety of other mammalian prey, such as bobcats (*Lynx rufus*), beavers (*Castor canadensis*), coyotes (*C. latrans*), porcupines (*Erethizon dorsatum*), and black bear cubs. Finally, the extra‐small prey (<7 kg/15 lbs) contained many species of birds, lagomorphs, rodents, red foxes (*Vulpes vulpes*), raccoons (*Procyon lotor*), and neonate mule deer from birth to 1 month old.

Similar studies focused on feeding rates of cougars have calculated biomass (kg) of prey killed per day in a set monitoring period (Elbroch et al., 2014; Knopff et al., 2010) or kill rate using an interkill interval or ratio estimator approach (Hebblewhite et al., 2013; Knopff et al., 2010). Due to the nature of our data, we chose to model the number of nights spent feeding at a kill or scavenge as the feeding bout duration, as identified by GPS‐collar data. This metric of handling time at a prey item is directly linked to energetic return to the cougar and is robust to any time lags in data collection by field crews. Additionally, this metric allows us to consider all prey items as food items without differentiating between predation events and scavenges.

We employed linear mixed‐effects modeling to explore the variation in feeding bout duration using the lmer() function from package lme4 in program R (v 3.5.1). Our response variable was the log of nights spent feeding on a prey item. We included cougar ID as a random intercept to control for individual cougar variation. We used 10 potential covariates to develop our model set: presence of any‐age‐dependent kittens, presence of kittens >3 months, prey weight class, cougar sex, local bear density, local deer density, days between kill and investigation, predation month, a binary covariate for local bear occupancy (absent or present), and a binary covariate for bear visitation (scavenging evidence at the prey item). Based on our knowledge of the system and other recent studies of cougar predation ecology, we developed a set of 44 hypothetical models that included combinations and interactions between the covariates of interest (Table S1). We evaluated these models using AIC to determine the top models that explain the variation in feeding bout duration by cougars. All models within <∆2 from the top model were considered to be supported by the data (Burnham & Anderson, 2002). Covariates included in the top models were interpreted as significant effects if they produced a *p*‐value less than an alpha of 0.05.

### 2.3 Prey composition

To examine the effects of bear recolonization on cougar prey composition in western Nevada, we used the same dataset of kill‐site investigations used in the feeding bout duration analysis. However, instead of classifying the prey items based on their approximated live weight, we separated the identified prey species into three taxonomic groups: mule deer, feral horses, and other (including domestic cattle, bighorn sheep, and nonungulate prey). We then calculated our response variable, the proportion of deer in cougar diet, which is the proportion of mule deer prey fed on out the total number of prey items located for each cougar in each year.

We fit generalized linear mixed models using the glmmTMB() function with a beta distribution from package glmmTMB in program R (v 3.5.1). The beta distribution was chosen because our response variable was bound between 0 and 1. We again included cougar ID as a random intercept to control for the variation among different individuals. We used 7 potential covariates to develop our model set: the presence of dependent kittens, the presence of kittens >3 months, cougar sex, local bear density, local deer density, days between kill and investigation, and a binary covariate for local bear occupancy. Based on our knowledge of the system and other recent studies of cougar predation ecology, we developed a set of 25 models that included combinations and interactions between the covariates of interest (Table S2).

Due to the differences in large prey availability between the Sierra Nevada and the Great Basin, we also divided the prey composition dataset into two regions. We fit the two regional datasets with the same set of 26 models using the same 7 covariates as described above. For the Sierra Nevada (Carson Range), 5 of the models would not converge because they only contained 1 factor of a binary covariate. For the Great Basin models, 1 of the models would not converge for the same reason. This resulted in 21 models fit for the Sierra Nevada (Table S3) and 25 models fit for the Great Basin (Table S4). We evaluated all models using AIC to determine the top models that explain the variation in feeding bout duration by cougars in the three separate datasets (overall, Sierra Nevada, Great Basin). All models identified as being < ∆2 from the top model were considered to be supported by the data (Burnham & Anderson, 2002).

To determine the direction of prey switching when alternative large prey is available, we modeled the proportion of horses in cougar diet for the Great Basin dataset only, since there are no feral horses in the Sierra Nevada. The response variable in this analysis was the proportion of feral horse prey fed on out of the total number of prey items located for each cougar in each year. We fit the set of 25 models to the Great Basin dataset as we did in the prior analysis (Table S5). We used AIC to rank the models, where models within < 2 AIC from the top model were considered supported by the data (Burnham & Anderson, 2002). Covariates included in the top models were interpreted as significant effects if they produced a *p*‐value less than an alpha of 0.05.

## 3 RESULTS

Global positioning system collars fitted on study animals had a 96.27% (± 3.17% *SD*) fix success rate, from which the kill‐site investigation dataset (used for both analyses) was extracted. The dataset included 884 confirmed prey items that were fed on by 31 cougars (10M:21F) between 2009–2012 and 2015–2017. Kill‐site investigations were conducted an average of 70 days after the cluster was formed. The length of days between the cluster formation date and investigation date was included as a potential linear predictor in both analyses but was not significant in either.

Habitat type where the kill or scavenge was located was derived from USGS GAP vegetation data and included as a covariate in both model sets. However, during model building, we observed that >80% of our kill‐site investigations were made in either sagebrush‐scrubland or pinyon‐juniper woodland, reflecting the predominant habitat types in the study site. These two major habitat types did not have a significantly different impact on either the feeding bout duration or the proportion of deer in the average cougar diet in initial data exploration, so habitat type was removed from the potential models due to being an uninformative parameter. Although habitat type is likely important in shaping cougar predation behavior and hunting success across the broad range of cougars, it was not particularly informative in this analysis.

### 3.1 Feeding bout duration

**FIGURE 2:**
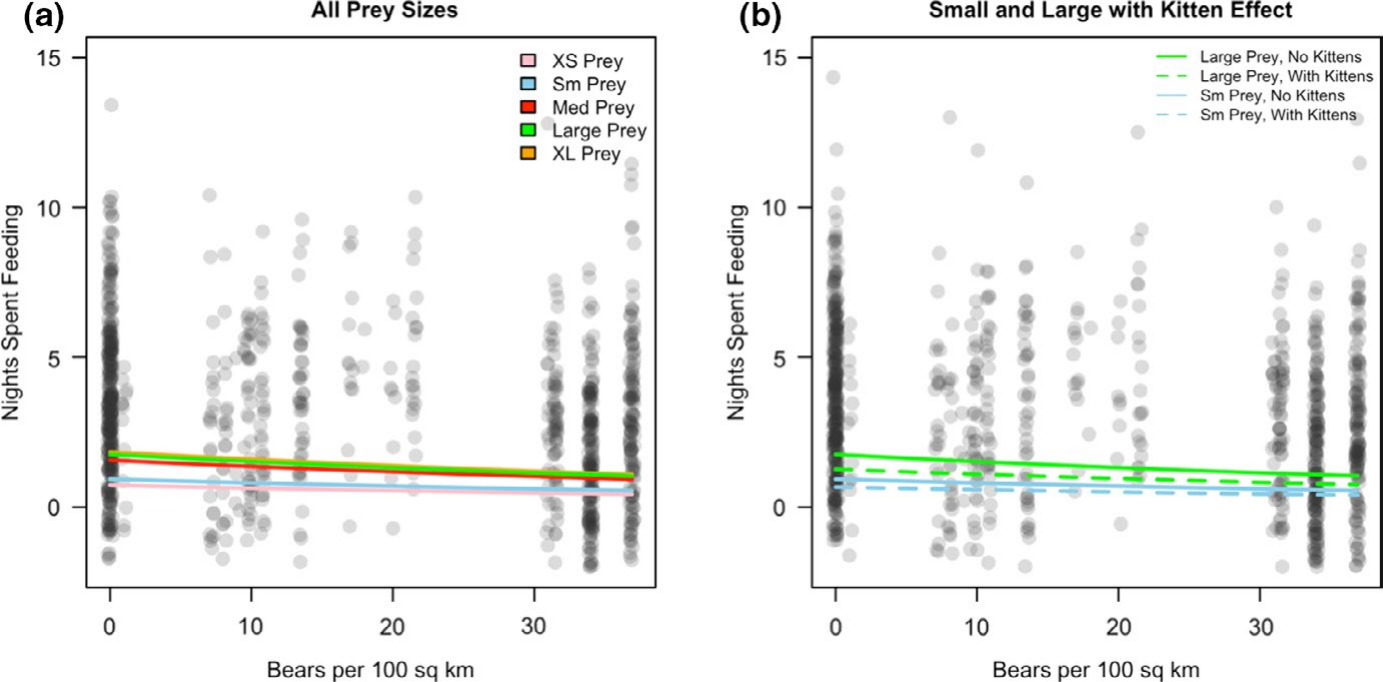
Feeding bout durations for cougars (*N* = 31) in the Great Basin and Sierra Nevada (combined), Nevada, USA, are predicted from the top model, where nights spent feeding at a prey item are a function of the weight class of the current prey item, local bear density, and the presence of dependent kittens. Data were collected between March and October 2009–2012 and 2015–2017. Panel A reflects the prediction of the model across all prey sizes, without illustrating the effect of dependent kittens. Panel B shows only the small and large prey but illustrates the effect of kitten presence in significantly reducing the feeding bout duration within each prey size class

Forty‐four hypothetical models were developed for predicting the feeding bout duration (in nights spent on a prey item) of cougars, with 1 top model identified. No other models were within < 2 Δ AIC of the best model (Table 2A, Table S1). The range of feeding bout durations at kills was from 1 to 24 nights spent, with a mean of 3.13 nights. The top model for the length of a feeding bout was a function of the weight class of the prey item, local bear density, and the presence of kittens >3 months old (Figure 2, Table S6). Nights spent on a prey item significantly increased with the increasing size of the prey item from the reference level of extra‐small prey (β_small_ = 0.24, *SE* = 0.13; β_med_ = 0.77, *SE* = 0.15; β_large_ = 0.88, *SE* = 0.12; β_x‐large_ = 0.93, *SE* = 0.15) and significantly decreased with both increasing bear density (β = −0.22, *SE* = 0.07) and the presence of kittens older than 3 months (β = −0.33, *SE* = 0.09).

**TABLE 2:** Top mixed models (Δ AIC < 2) for (A) for feeding bout duration (in nights spent on a prey item) and (B) deer in diet composition in all sites, (C) deer in diet composition in the Sierra Nevada, (D) deer in diet composition in the Great Basin, and (E) horse in diet composition in the Great Basin. All models use kill‐site data on clusters formed between March and October of 2009–2012 and 2015–2017. Only the top model of 10 models is shown for B. See Supporting Information for details on the other 9 models

	*df*	AIC	Δ AIC	K
A. Feeding bout duration—Great Basin and Sierra Nevada				
Nights Spent ~ Prey Weight Class + Bear Density + Kittens (3 months) + (1 | Cougar ID)	9	2,308.45	0	7
B. Prey composition—Great Basin and Sierra Nevada				
Proportion Deer in Diet ~ Bear Density + Cougar Sex + Year + (1 | Cougar ID)	6	−3,049.99	0	4
C. Prey composition—Sierra Nevada only				
Proportion Deer in Diet ~ Bear Density * Cougar Sex + Kittens (any‐age) + (1 | Cougar ID)	7	−1,721.70	0	5
D. Prey composition—Great Basin only				
Proportion Deer in Diet ~ Bear Density + Cougar Sex + Year + (1 | Cougar ID)	6	−1,449.55	0	4
Proportion Deer in Diet ~ Bear Density + Year + (1 | Cougar ID)	5	−1,448.77	0.775	3
Proportion Deer in Diet ~ Bear Density * Cougar Sex + Year + (1 | Cougar ID)	7	−1,447.59	1.954	5
E. Prey composition—Horses in Great Basin only				
Proportion Feral Horse in Diet ~ Year + Cougar Sex + Dependent Kittens + (1 | Cougar ID)	6	−2,996.72	0	4
Proportion Feral Horse in Diet ~ Bear Density + Year + Dependent Kittens + (1 | Cougar ID)	6	−2,995.37	1.35	4
Proportion Feral Horse in Diet ~ Year + Cougar Sex + Dependent Kittens + Deer Density + (1 | Cougar ID)	7	−2,995.02	1.7	5

### 3.2 Prey composition

**FIGURE 3:**
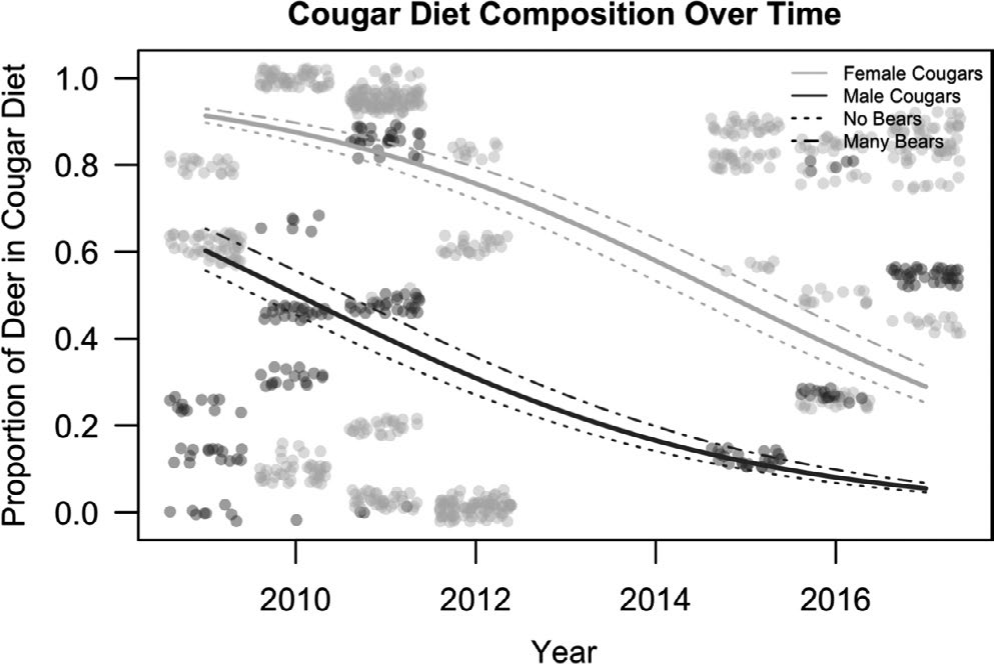
The proportion of deer in the average cougar's diet in Nevada's Great Basin and Sierra Nevada, USA, predicted as a function of year, bear density, and cougar sex. Each gray circle represents one kill‐site investigation, with dark gray points and lines indicating male cougar data and light gray points and lines indicating female cougar data. Prediction lines are plotted for males and females at the average bear density (solid lines), at the maximum bear density (dash‐dot line), and at the minimum bear density, which illustrates no bears (dotted line)

Twenty‐five hypothetical models were developed for predicting the proportion of mule deer in cougar diets in Nevada. Ten models were identified within <2 Δ AIC units of the top model (Table 2B, Table S2). The top model was thus interpreted as the model with the smallest AIC, which included three significant covariates: a positive effect of bear density (β = 0.17, *SE* = 0.08), and a negative effect of male cougars (β = −1.94, *SE* = 0.95) and year (β = −0.41, *SE* = 0.05; Figure 3, Table S7a). This set of 10 competing models also included one model that was more parsimonious (had fewer degrees of freedom) than the top model, but the more parsimonious model only differed by the exclusion of the cougar sex parameter (Table S2). Because cougar sex was significant in the top model (*p* < .05), we retained the cougar sex covariate in the top model for interpretation (Table 2, Figure 3).

**FIGURE 4:**
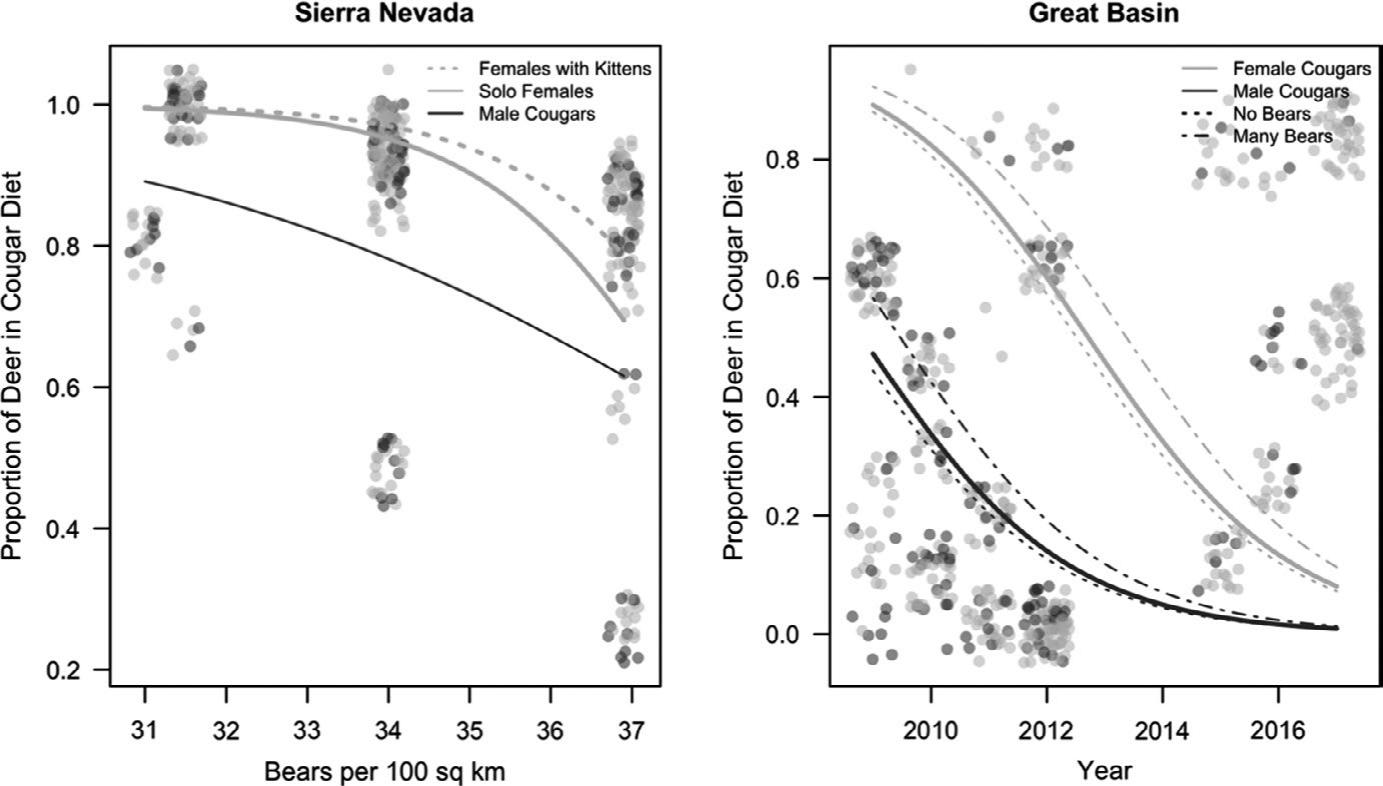
The proportion of deer in the average cougar's diet in Nevada's Sierra Nevada and Great Basin, USA. In panel A, the proportion of deer in the diet of cougars in the Sierra Nevada is predicted as a function of local bear density, cougar sex, and the presence of dependent kittens. In panel B, the proportion of deer in the diet of cougars in the Great Basin is predicted as a function of year, cougar sex, and local bear density. Each gray circle represents one kill‐site investigation, with dark gray points and lines indicating male cougar data and light gray points and lines indicating female cougar data

We also analyzed the Sierra Nevada (*n* = 362 prey items) and the Great Basin (*n* = 552 prey items) prey composition datasets separately using the same set of hypothesized models, except for six models that did not converge. One model was identified (with no competing models within <2 Δ AIC) for the Sierra Nevada (Table 2C, Table S3). This model included the significant negative effects of local bear density (β = −11.45, *SE* = 1.03) and male cougars (β = −9.56, *SE* = 1.83), and the significant positive effects of the presence of any‐age‐dependent kittens (β = 0.48, *SE* = 0.11) and interaction between bear density and male cougars (β = 7.19, *SE* = 1.51; Figure 4a, Table S7B). Three top models were identified for the Great Basin dataset with < 2 Δ AIC (Table 2D, Table S4). The top model included the significant positive effect of local bear density (β = 0.35, *SE* = 0.16), the significant negative effect of year (β = −0.57, *SE* = 0.08), and a nonsignificant negative effect of male cougars (β = −2.22, *SE* = 1.3, *p* = .88; Figure 4b, Table S7c). The second‐best model dropped the cougar sex parameter, and the third‐best model included an additional covariate of the interaction between bear density and male cougars (Table S7c).

**FIGURE 5:**
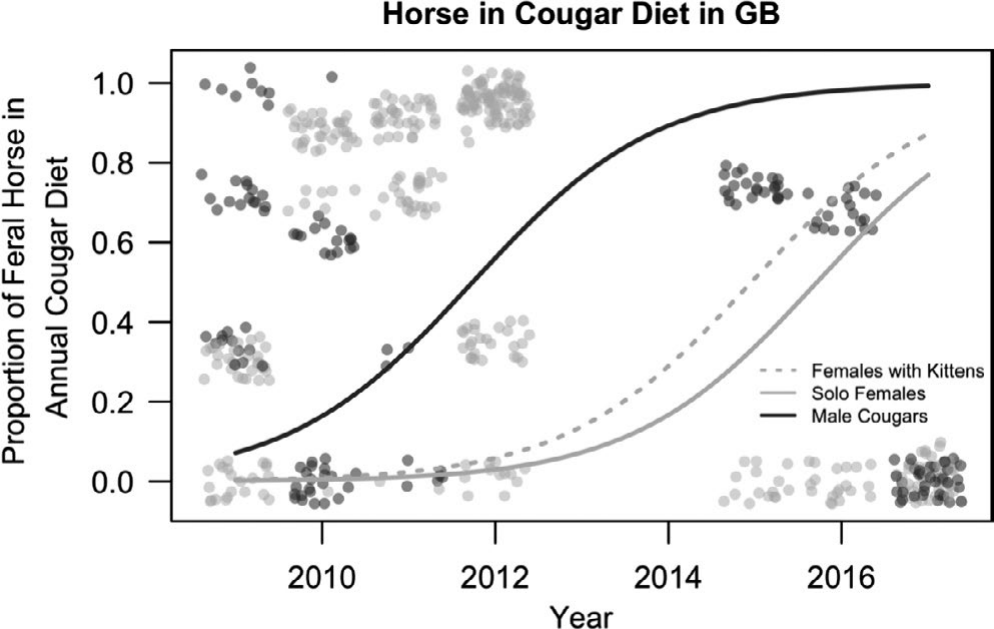
The proportion of feral horse in the average cougar's diet in Nevada's Great Basin, USA. The proportion of feral horse in the diet is predicted as a function of year, cougar sex, and the presence of dependent kittens. Each gray circle represents one kill‐site investigation, with dark gray points and lines indicating male cougar data and light gray points and lines indicating female cougar data

For the proportion of horses in the diet for the Great Basin dataset only, we identified 3 top models that were within <2 Δ AIC (Table 2E, Table S5). The top model included the significant positive effects of year (β = 0.94, *SE* = 0.07) and the presence of dependent kittens (β = 0.71, *SE* = 0.13) and a nonsignificant positive effect of cougar sex (male cougars β = 3.73, *SE* = 2.37, *p* = .11; Figure 5, Table S7d). The second‐best model added a nonsignificant negative effect for increased bear density (β = −0.18, *SE* = 0.18, *p* = .32) in place of the cougar sex parameter (Table S7d). The third‐best model retained the cougar sex parameter, year, and presence of kittens from the top model, but added the nonsignificant positive effect of deer density (β = 0.003, *SE* = 0.06, *p* = .58; Table S7D). In models 2 and 3, bear density and deer density were not significant (*p* = .324 and *p* = .582, respectively), whereas year was significant (*p* = 2 × 10^−16^ for both) in both models. Thus, we interpret the top and simplest model as the best fit model for this dataset (Table 2, Figure 5).

## 4 DISCUSSION

Our analyses show the importance of considering multiple factors that may influence the foraging behavior of a large carnivore when assessing the influence of a recolonizing intraguild competitor. Although there has been thorough documentation of how recolonizing wolf populations impact cougar predation behavior (Atwood et al., 2007; Orning, 2019), there has been relatively little work addressing changes in cougar predation behavior with increasing or recolonizing black bear populations (but see Ruth & Buotte, 2007). Further, there is a dearth of literature from across the globe on the impacts of recolonizing dominant carnivores that also scavenge on prey killed by naïve subordinate carnivore species. This is especially true outside of protected areas. This knowledge gap is critical to fill as many of the world's large carnivores scavenge and kill prey and live outside of protected areas. Our data add to this important area of inquiry.

Our results show that the most important variables driving cougar feeding duration during the time of year when bears are also active include the size of the current prey item, bear density, and kitten presence. The presence of young kittens significantly reduced the duration of feeding bouts, similar to what has been found in other studies (Knopff et al., 2010; Tallian et al., 2017). Importantly, we found that cougar feeding durations on prey items were significantly shorter in areas of high bear densities. Cougar sex, local bear density, and the presence of dependent kittens were significant predictors of cougar prey composition. Importantly, the most prominent driver of cougar prey composition in three of our four analyses was year. This highlights the importance of collecting data over a longer period in a dynamic system. In each model, the effect of year was larger and had a smaller standard error than the effect of bear density, so we considered year to be the primary predictor in this system. The percent of deer in cougar diet significantly declined over time (across years) for all scenarios tested, with a tight 95% confidence interval. Female cougars had a higher proportion of deer in their diet than male cougars across all years, although the 95% confidence intervals for these estimates were larger and crossed 0 in some models. The presence of dependent kittens significantly increased the proportion of deer in female cougar diet in all top models where it was included. The proportion of deer in the diet was higher at high bear densities and was lower in sites with no bears across all models where bear density was included. The 95% confidence intervals for bear density were wide in some models and approached 0, but did not cross 0 for any model in which bear density was included.

In the top model for feeding bout duration, handling time at a prey item logically decreased as the weight class of the prey item decreased and decreased further within each prey weight class if a female cougar had dependent young (Figure 2). This result corroborates previous research where mothers with dependent young had the highest kill rates (in terms of both biomass per day and kills per week) of all demographic groups and, consequently, the shortest interkill intervals, likely to meet the nutritional needs of their dependent young (Clark et al., 2014; Elbroch et al., 2015; Knopff et al., 2010).

The effect of bear density was also significant in the feeding bout duration model. Our model offers support for our hypothesis that increased local bear density is associated with shorter cougar feeding durations on each food item (i.e., fewer nights spent at prey; Figure 2). Recolonizing bear presence and increasing bear density over time may result in more bear encounters that force cougars to leave their prey before they have completely depleted the carcass. In our system, both bear densities and bear scavenging events have increased over time in sites with recolonizing black bears (Table 1, Figure S1, Figure S2). Increased cougar kill rates as a result of bear displacement have been documented in other studies in western North America (Elbroch et al., 2015). Due to the time lag between cluster formation and kill‐site investigation in our dataset, we were largely unable to differentiate between passive scavenging, where bears feed on the remains of a kill that a cougar has already abandoned, and kleptoparasitism, where bears actively displace a cougar from the prey resource. Thus, our predictor of bear presence on a kill was not significant in explaining the feeding bout duration. However, the relationship of bear density to cougar feeding bout duration suggests that cougars were actively displaced from their kills or that they perceived a higher risk of displacement due to the increased local bear density and chose to abandon their prey sooner. Cougars in areas with higher bear densities spent fewer nights feeding on a given prey item, which may require them to hunt again more quickly to fulfill their energetic needs. In this way, dominant scavengers may shift the predation behavior of solitary predators, leading to increased kill rates of either primary or alternative prey (Elbroch et al., 2015; Krofel et al., 2012).

Our top model for cougar prey composition in the overall dataset in Nevada indicated that year was significantly correlated with a decreasing proportion of deer in both male and female cougar diet, but that female cougars had a significantly higher proportion of deer in their diets from March to October in all years relative to male cougars (Figure 3). Although our confidence interval for the effect of cougar sex did not overlap 0, it was wide relative to the effect of year. However, our results are similar to other studies in which females tend to prey primarily on mule deer and other medium‐sized prey, and males tend to feed on the larger‐bodied prey available in the system (feral horses in our current study, elk and/or moose in Clark et al., 2014; Knopff et al., 2010). Interestingly, the opposite has been observed in female cougars sympatric with recolonizing wolf populations; female cougars living with sympatric wolves decreased their usage of deer compared to female cougars in the same region before wolf recolonization (Orning, 2019). However, pack hunting by wolves likely influences cougar behavior in different ways than scavenging by solitary bears and may explain this phenomenon.

Bear density also had a significant effect on the proportion of deer in the diet across all years, with high bear densities shifting the model prediction upwards. For sites without recolonized bears, this prediction line was shifted slightly downwards (Figures 3 and 4). These models indicate that our sample of female cougars incorporated deer into their diet at a higher rate throughout the season when bears are active (March through October) and at all bear densities relative to our sample of male cougars, who instead incorporated a higher proportion of feral horses into their diet (Figure 5). However, Andreasen et al., (2021) demonstrated that, early in the Great Basin study area when bear densities were lower, female cougars relied more heavily on feral horses throughout the winter months compared with male cougars. While seemingly contradictory to our results, when combined, these results suggest that females are fully capable of killing feral horses year‐round but may primarily do so in the winter when the risk of kleptoparasitism is lower. Alternatively, our data were collected over a longer period of years and may illustrate how foraging patterns of cougars can change alongside changes in bear density as cougars may learn new strategies to respond to the presence of bears during the time of each year that both are active. This may explain why we found that male cougars take a significantly higher proportion of feral horses over mule deer from March to October. Further, we detected individuals with explicit mule deer‐dominated or horse‐dominated diets, which could drive changes in population‐level patterns across time as the cougar population turns over (see Andreasen et al., 2021).

Individual cougars can show extreme specialization (Lowrey et al., 2016). In fact, individual variation was important in determining the prey composition of cougars in our study, as several cougars readily preyed on feral horses when both horses and deer were available. Of the eight males in our Great Basin prey composition analysis, two individuals had diets dominated by feral horses, with 67.5% (27/40) and 73% (11/15) of their annual diet composed of horses. Four of the 13 females in the Great Basin prey composition analysis had diets dominated by horses. These four females had 78% (25/32), 86.4% (70/81), 89.2% (33/37), and 91.7% (33/36) of their diet composed of horses over other available prey types. Interestingly, female cougars who selected for feral horses ate a higher proportion of horse in their diet (78%–91.7%) than males that specialized on horses (67.5%–73%). The remaining six males and nine females had diets dominated by mule deer, showing that individual variation and preference in prey composition can widely vary in the same population. Although certain female and male cougars specialized in killing horses, this effect was moderated when looking at population‐level metrics. In this study, we were primarily concerned with the overall trends in prey composition at the population level during the spring and summer to focus on the influence of bear activity on average cougar foraging behavior. Other studies focusing on the ecological drivers behind individual prey selection and consumption rates (e.g., Balme et al., 2020) could be relevant to the role of horses in the diet of cougars in this system (Andreasen et al., 2021).

Individual prey selection also influenced the results of models comparing the Sierra Nevada and Great Basin ranges. In these prey composition models, we see how cougars respond differently to competition from bears depending on whether they live in a region with low prey diversity and high bear density (Sierra Nevada) or a region with high prey diversity but lower bear densities (Great Basin). In the Sierra Nevada, male and female cougars at the highest bear densities (30–40 bears per 100 km^2^) converge on feeding primarily on mule deer (about 80%–100%). This is representative of the typical single‐predator, single‐prey system that we often see with cougars and mule deer throughout their range in the intermountain West. The declining proportion of deer in male cougar diet at the highest bear densities in the Sierra Nevada (Figure 4a) is driven primarily by one individual male who consumed a diet of 30% beavers during the study. Excluding this individual, 87% of the kills in the Sierra Nevada were of mule deer, which indicates that this individual male's selection for beavers drove the observed pattern. Mule deer represent an optimal prey size for cougars where the risk of injury during an attack is low, and cougars can consume a sufficient amount of the prey resource before potentially being displaced by a bear.

In the Great Basin, year was the best predictor of the proportion of deer in the diet (Figure 4b). Cougars can also select feral horses, bighorn sheep, pronghorn, and domestic cattle in addition to the mule deer and nonungulate prey found in the Sierra Nevada. As Andreasen et al., (2021) also found, both sexes consume a lower proportion of deer in the Great Basin than in the Sierra Nevada (Figure 4b). The proportion of mule deer in the diet was higher for females than males at all times, but both sexes demonstrated a significant decline in foraging rates on mule deer, and a concurrent increase in foraging rates on feral horses, in the later years of the study (Figure 4b, Figure 5). For each sex across time, high bear densities significantly increased the proportion of deer in the diet, and cougars in the area with no recolonized bears had a decreased proportion of deer in their diet (Figure 4b). This indicates that cougars may respond to increased bear density (which likely results in displacement from their kills) by making additional deer kills to supplement caloric losses, although this hypothesis should be directly tested. Due to the diversity of prey and availability of horse populations to feed on, prey selection by cougars in the Great Basin appears to be influenced by a complex array of factors.

Overall, our model results suggest that cougar predation behavior is changing over time in the Sierra Nevada and Great Basin ranges. Specifically, cougars are responding to growing bear density, and a likely increase in scavenging pressure, by spending fewer nights feeding at each prey item, supporting our first hypothesis. In the Sierra Nevada, where smaller mammalian prey is the only alternative to mule deer, our data indicate that cougars experiencing the highest bear density (and likely the highest risk of bear scavenging) have a higher proportion of nonungulate mammals in their diet than cougars at lower bear densities, supporting our second hypothesis. However, in the Great Basin, the proportion of deer in the also diet declined over time, but both male and female cougars increasingly utilized feral horses (Figure 5) instead of small mammalian prey as we hypothesized. Additionally, in each year, cougars in areas with higher bear densities fed more heavily on mule deer than cougars in areas with no bears.

Bear and deer densities exceeded our correlation threshold of 0.7, and it is likely that high‐quality habitat for mule deer also provides high‐quality food resources for bears, in terms of the available mast, vegetation, insects, neonate deer fawns, and carrion from both natural mortalities and cougar‐killed deer (Mitchell & Powell, 2007). For this reason, we did not include these two covariates in the same hypothesized models but evaluated each covariate separately within the set of potential models. Our top models suggested that bear density was much more informative than deer density in predicting both the length of a feeding bout and the proportion of deer in cougar diet.

In the mountain ranges with resident bears in our study, bear density was steadily increasing over time (Beckmann & Lackey, 2018). The increasing local bear density (Figure S1) and the increased bear scavenging of cougar kills (Figure S2) may lead to a shift in the composition of cougar diet and the length of time individual cougars feed on each prey item. Compared to feral horses, mule deer are a safer and easier prey item for cougars to subdue, but they provide less consumable biomass. In sites where bears have recolonized, 44% of all mule deer kills were scavenged by bears, compared with 23% of feral horses or 24% of other mammalian prey. Cougars in areas with low bear densities likely have a lower risk of losing their kills to bear scavenging, which may increase their willingness to kill larger and more dangerous ungulates. In areas with high bear density, the risk of injury to a cougar while killing a horse may outweigh the foraging benefit gained if bears frequently kleptoparasitize kills from cougars, leading cougars to select for the smaller, safer prey species (i.e., deer) where the risk of losing prey resources is consistently high.

Interference competition, including kleptoparasitism, causes loss of prey resources and search time from the subordinate competitor and has been documented in many systems (Krofel et al., 2012; Murphy et al., 1998; Tallian et al., 2017). Several previous studies have documented evidence that kleptoparasitism forces the subordinate carnivore to abandon their kill and increase their kill rate to compensate for the lost biomass (Elbroch et al., 2015; Elbroch & Wittmer, 2013; Krofel et al., 2012). However, recent work has shown that the impact of scavengers on carnivores may not always be straightforward. Tallian et al., (2017) found that the presence of brown bears (*Ursus arctos*), another dominant scavenger, lengthened the interkill intervals (i.e., decreased kill rates) of wolf packs across two distinct systems. Similarly, Orning (2019) found that cougars sympatric with recolonizing gray wolves had reduced kill rates, lower biomass consumption rates, and reduced proportions of mule deer in their diet compared with cougars in the same area before wolf recolonization. In the current study, the feeding bout duration decreased and the proportion of deer in cougar diet increased with higher bear density, which appears consistent with the hypothesis that interference competition from a growing bear population may drive predatory behavioral changes in cougars in Nevada. Certainly, the diet of cougars has changed over time in this region, as evidenced by the significance of year in several models. A decrease in feeding bout duration (and the probable correlated increase in kill rate) comes at an energetic cost for cougars, and these novel competitive interactions may reduce cougar reproductive success, individual fitness, and population growth (Elbroch et al., 2015; Orning, 2019). At the same time, increases in cached food subsidies for black bears made available by cougars may have assisted the rapid recolonization of bears in the Great Basin, compared to if food subsidies provided by cougars had been unavailable (Beckmann & Berger, 2003), an idea that deserves further investigation in this system. The diverse responses of individual predators and predator–scavenger guilds to competition and scavenging pressure suggest that the composition of each predator community may influence the results of the resource competition and, thus, also warrants additional attention.

While our findings suggest that the foraging behavior of cougars is impacted by black bears, we could not fully tease apart the competitive relationship between these two carnivores and the subsequent impact on the community because we were unable to differentiate active kleptoparasitism from passive scavenging after the cougar has left on its own accord. Despite this limitation, our study identifies that cougar feeding bout durations in Nevada's Great Basin and Sierra Nevada are primarily driven by the size of the prey item most recently consumed, local bear density, and the presence of dependent kittens with a female. Diet composition changed over time, with the proportion of mule deer in the diet generally decreasing over time in all sites. However, in sites with high prey diversity (Great Basin), higher bear densities were associated with an increased proportion of mule deer in cougar diet, but in low prey diversity sites (Sierra Nevada), the highest bear densities were associated with reduced proportions of mule deer in the diet. Cougar sex and the presence of dependent young also influenced cougar diet composition in this highly complex system. We conclude that the growth of recolonizing bear populations over time and the subsequent increased scavenging pressure during the spring and summer may alter cougar predation behavior in Nevada as cougars respond to this novel competition pressure. In the context of species recolonization and naïve competitors, we expect that increasing contact between a caching apex predator and a dominant carnivore–scavenger may lead to behavioral changes, as well as changes in growth rates, in one or both species. For example, cougars may lose food resources and thus have to allocate more time and energy to increased hunting efforts, and bears may be able to recolonize areas more quickly due to carrion food subsidies than they could in areas without cougars. This look into a dynamic natural recolonization system allows us to begin to document the range of intraguild behavioral responses to novel resource competition.

Nevada's Sierra Nevada and Great Basin provide a unique opportunity to determine the impacts of large carnivore and scavenger recoveries that are happening across the globe due to recent conservation efforts (e.g., see Bartnick et al., 2013; Beckmann & Lackey, 2018; Harihar et al., 2011). In this system, a once‐extirpated dominant carnivore that is a facultative scavenger has returned in numerical and functional ways, thus usurping another carnivore that had functioned as the apex predator for nearly a century. The resulting changes in predator–scavenger and predator–prey interaction processes we describe here are likely to occur in other working landscapes across the globe. These findings can offer unique insight into the impacts of large carnivore and scavenger recoveries on the trajectory of resident intraguild predators in systems outside of protected areas.

The growth or expansion of a predator species may have unexpected effects on previously established populations of intraguild competitors, such as inducing diet shifts or altering population demographic rates (Bartnick et al., 2013; Harihar et al., 2011; Orning, 2019). Additionally, predators can have a variety of impacts on their prey species, many of which are not fully understood (Ford and Goheen 2015, Winnie & Creel, 2017). Although large carnivores may ignite controversy and sociopolitical tension, some regions have seen successful recoveries of large carnivores and their prey despite high human population densities (Chapron et al., 2014). Understanding the full impacts of large predator restoration is an important next step in making conservation and management decisions at local and landscape levels, as growing human‐dominated ecosystems continue to be managed to recover missing taxa over the next several decades.

## CONFLICT OF INTEREST

The authors have no conflicts of interest to declare.

## AUTHOR CONTRIBUTIONS

**Kristin Engebretsen:** Conceptualization (supporting); Data curation (supporting); Formal analysis (lead); Writing‐original draft (lead); Writing‐review & editing (equal). **Jon Beckmann:** Conceptualization (equal); Data curation (equal); Funding acquisition (lead); Methodology (equal); Project administration (equal); Resources (supporting); Supervision (equal); Writing‐original draft (supporting); Writing‐review & editing (equal). **Carl Lackey:** Conceptualization (equal); Data curation (supporting); Funding acquisition (equal); Project administration (equal); Resources (lead); Writing‐original draft (supporting); Writing‐review & editing (equal). **Alyson Andreasen:** Conceptualization (equal); Data curation (equal); Funding acquisition (equal); Methodology (equal); Project administration (equal); Writing‐original draft (supporting); Writing‐review & editing (equal). **Cody Schroeder:** Data curation (supporting); Project administration (supporting); Resources (supporting); Writing‐review & editing (supporting). **Pat Jackson:** Project administration (supporting); Resources (supporting); Writing‐review & editing (supporting). **Julie Young:** Conceptualization (supporting); Formal analysis (supporting); Funding acquisition (supporting); Methodology (supporting); Project administration (equal); Resources (supporting); Supervision (equal); Writing‐original draft (equal); Writing‐review & editing (equal).

## Supporting information

Supplementary Material

## Data Availability

The data underlying this article are available at Dryad, https://doi.org/10.5061/dryad.9ghx3ffg.
