# Supplementary material for: Recolonizing carnivores: Is cougar predation behaviorally mediated by bears?
Source: Ecol Evol. 2021 Mar 21;11(10):5331–43. doi: 10.1002/ece3.7424 (PMC8131799; doi:10.1002/ece3.7424)
Supplement: Supplementary file 1 — Supplementary Material [file ECE3-11-5331-s001.docx]

**Suppl. Table 1.** The set of 44 possible linear mixed models to predict cougar feeding bout duration (number of nights spent on a prey item) in Nevada’s Great Basin and Sierra Nevada Range between 2009-2012 and 2015-2017. All models were fit with one random intercept, Cougar ID, to control for the variation across individuals.

| **Model Name** | **df** | **AIC** | **deltaAIC** |
| --- | --- | --- | --- |
| **Nights Spent ~ Prey Weight + Bear Density + Kittens (> 3 months) + (1 \| CougarID)** | **9** | **2308.435** | **0** |
| Nights Spent ~ Prey Weight + Bear Density + Kittens (> 3 months) + Year + (1 \| CougarID) | 10 | 2312.927 | 4.492 |
| Nights Spent ~ Prey Weight + Bear Density + (1 \| CougarID) | 8 | 2316.228 | 7.793 |
| Nights Spent ~ Prey Weight + Bear Density + Bear Visit + (1 \| CougarID) | 9 | 2317.362 | 8.927 |
| Nights Spent ~ Prey Weight + Bear Density + Kittens (Any Age)+ Year + (1 \| CougarID) | 10 | 2318.826 | 10.391 |
| Nights Spent ~ Prey Weight + Kittens (> 3 months) + Year + (1 \| CougarID) | 9 | 2318.986 | 10.551 |
| Nights Spent ~ Prey Weight + Bear Visit + Kittens (> 3 months) + Year + (1 \| CougarID) | 10 | 2319.695 | 11.26 |
| Nights Spent ~ Prey Weight + Bear Density + Year + (1 \| CougarID) | 9 | 2321.426 | 12.991 |
| Nights Spent ~ Prey Weight + (1 \| CougarID) | 7 | 2321.548 | 13.113 |
| Nights Spent ~ Prey Weight + Bear Density + Bear Visit + Year + (1 \| CougarID) | 10 | 2322.365 | 13.93 |
| Nights Spent ~ Prey Weight + Bear Occupancy + Bear Visit * Kittens (> 3 months) + Year + (1 \| CougarID) | 12 | 2325.431 | 16.996 |
| Nights Spent ~ Prey Weight + Kittens (Any Age)+ Year + (1 \| CougarID) | 9 | 2325.725 | 17.29 |
| Nights Spent ~ Prey Weight + Bear Visit + Kittens (Any Age) + Year + (1 \| CougarID) | 10 | 2325.867 | 17.432 |
| Nights Spent ~ Prey Weight + Bear Visit + Year + (1 \| CougarID) | 9 | 2327.954 | 19.519 |
| Nights Spent ~ Prey Weight * Cougar Sex + Bear Density + Year + (1 \| CougarID) | 14 | 2328.277 | 19.842 |
| Nights Spent ~ Prey Weight + Year + (1 \| CougarID) | 8 | 2328.591 | 20.156 |
| Nights Spent ~ Prey Weight + Bear Density * Cougar Sex + Year + (1 \| CougarID) | 11 | 2329.245 | 20.81 |
| Nights Spent ~ Prey Weight + Bear Visit * Bear Occupancy + Year + (1 \| CougarID) | 11 | 2330.263 | 21.828 |
| Nights Spent ~ Prey Weight + Bear Occupancy + Bear Visit + Year + (1 \| CougarID) | 10 | 2330.977 | 22.542 |
| Nights Spent ~ Prey Weight + Bear Occupancy + Year + (1 \| CougarID) | 9 | 2331.280 | 22.845 |
| Nights Spent ~ Prey Weight + Cougar Sex + Year + (1 \| CougarID) | 9 | 2331.779 | 23.344 |
| Nights Spent ~ Prey Weight + Bear Occupancy + Bear Visit * Kittens (Any Age) + Year + (1 \| CougarID) | 12 | 2332.243 | 23.808 |
| Nights Spent ~ Prey Weight + Deer Density+ Bear Visit + Year + (1 \| CougarID) | 10 | 2332.430 | 23.995 |
| Nights Spent ~ Prey Weight + Deer Density+ Year + (1 \| CougarID) | 9 | 2332.894 | 24.459 |
| Nights Spent ~ Prey Weight * Bear Density + Year + (1 \| CougarID) | 13 | 2333.109 | 24.674 |
| Nights Spent ~ Prey Weight + Bear Visit * Cougar Sex + Year + (1 \| CougarID) | 11 | 2333.953 | 25.518 |
| Nights Spent ~ Prey Weight + Deer Density + Bear Occupancy * Bear Visit + Year + (1 \| CougarID) | 12 | 2335.149 | 26.714 |
| Nights Spent ~ Prey Weight + Deer Density + Bear Occupancy + Year + (1 \| CougarID) | 10 | 2336.199 | 27.764 |
| Nights Spent ~ Prey Weight + Bear Occupancy * Cougar Sex + Year + (1 \| CougarID) | 11 | 2336.976 | 28.541 |
| Nights Spent ~ Prey Weight * Cougar Sex + Year + (1 \| CougarID) | 13 | 2337.042 | 28.607 |
| Nights Spent ~ Prey Weight + Days Between Kill and Investigation + Year + (1 \| CougarID) | 9 | 2339.396 | 30.961 |
| Nights Spent ~ Prey Weight + Bear Occupancy * Cougar Sex + Deer Density + Year + (1 \| CougarID) | 12 | 2341.951 | 33.516 |
| Nights Spent ~ Prey Weight + Predation Month + Kittens (> 3 months) + Year + (1 \| CougarID) | 16 | 2343.717 | 35.282 |
| Nights Spent ~ Prey Weight + Predation Month + Year + (1 \| CougarID) | 15 | 2354.183 | 45.748 |
| Nights Spent ~ Prey Weight + Predation Month + Bear Visit + Year + (1 \| CougarID) | 16 | 2355.530 | 47.095 |
| Nights Spent ~ Bear Density + Year + (1 \| CougarID) | 5 | 2386.552 | 78.117 |
| Nights Spent ~ Kittens (> 3 months) + Year + (1 \| CougarID) | 5 | 2389.606 | 81.171 |
| Nights Spent ~ Year + (1 \| CougarID) | 4 | 2391.933 | 83.498 |
| Nights Spent ~ Kittens (Any Age)+ Year + (1 \| CougarID) | 5 | 2393.204 | 84.769 |
| Nights Spent ~ Bear Occupancy + Year + (1 \| CougarID) | 5 | 2394.455 | 86.02 |
| Nights Spent ~ Cougar Sex + Year + (1 \| CougarID) | 5 | 2394.742 | 86.307 |
| Nights Spent ~ Bear Visit + Year + (1 \| CougarID) | 5 | 2395.617 | 87.182 |
| Nights Spent ~ Deer Density+ Year + (1 \| CougarID) | 5 | 2396.999 | 88.564 |
| Nights Spent ~ Days Between Kill and Investigation + Year + (1 \| CougarID) | 5 | 2405.980 | 97.545 |

**Suppl. Table 2.** The set of 26 possible beta mixed models to predict the proportion of deer in the average cougar’s diet in Nevada’s Great Basin and Sierra Nevada between 2009-2012 and 2015-2017. All models were fit with one random intercept, Cougar ID, to control for the variation in individuals.

| **Model Name** | **df** | **AIC** | **deltaAIC** |
| --- | --- | --- | --- |
| **Proportion(Deer) ~ Bear Density + Cougar Sex + Year + (1 \| CougarID)** | **6** | **-3049.994** | **0** |
| **Proportion(Deer) ~ Bear Density + Cougar Sex + Kittens (Any Age) + Year + (1 \| CougarID)** | **7** | **-3049.882** | **0.112** |
| **Proportion(Deer) ~ Bear Density * Cougar Sex + Year + (1 \| CougarID)** | **7** | **-3049.109** | **0.885** |
| **Proportion(Deer) ~ Bear Density * Bear Present at Kill + Kittens (Any Age) + Year + (1 \| CougarID)** | **8** | **-3049.034** | **0.96** |
| **Proportion(Deer) ~ Bear Density * Cougar Sex + Kittens (>3 months) + Year + (1 \| CougarID)** | **8** | **-3048.873** | **1.121** |
| **Proportion(Deer) ~ Bear Density * Cougar Sex + Kittens (Any Age) + Year + (1 \| CougarID)** | **8** | **-3048.732** | **1.262** |
| **Proportion(Deer) ~ Deer Density + Cougar Sex + Kittens (Any Age) + Year + (1 \| CougarID)** | **7** | **-3048.364** | **1.63** |
| **Proportion(Deer) ~ Bear Density + Year + Kittens (Any Age) + (1 \| CougarID)** | **6** | **-3048.134** | **1.86** |
| **Proportion(Deer) ~ Bear Occupancy * Cougar Sex + Deer Density + Year + (1 \| CougarID)** | **8** | **-3048.097** | **1.897** |
| **Proportion(Deer) ~ Bear Density + Year + (1 \| CougarID)** | **5** | **-3048.063** | **1.931** |
| Proportion(Deer) ~ Cougar Sex + Kittens (>3 months) + Year + (1 \| CougarID) | 6 | -3047.920 | 2.074 |
| Proportion(Deer) ~ Cougar Sex + Year + (1 \| CougarID) | 5 | -3047.853 | 2.141 |
| Proportion(Deer) ~ Bear Occupancy * Cougar Sex + Kittens (Any Age) + Year + (1 \| CougarID) | 8 | -3047.486 | 2.508 |
| Proportion(Deer) ~ Deer Density + Year + (1 \| CougarID) | 5 | -3047.043 | 2.951 |
| Proportion(Deer) ~ Cougar Sex + Kittens (Any Age) + Year + (1 \| CougarID) | 6 | -3046.954 | 3.04 |
| Proportion(Deer) ~ Deer Density * Cougar Sex + Kittens (Any Age) + Bear Occupancy + Year + (1 \| CougarID) | 9 | -3045.287 | 4.707 |
| Proportion(Deer) ~ Bear Occupancy + HorsePresence + Kittens (Any Age) + Year + (1 \| CougarID) | 7 | -3043.948 | 6.046 |
| Proportion(Deer) ~ Bear Density + Kittens (Any Age) + Cougar Sex + (1 \| CougarID) | 6 | -2989.766 | 60.228 |
| Proportion(Deer) ~ Bear Density + Cougar Sex + Kittens (Any Age) + (1 \| CougarID) | 6 | -2989.766 | 60.228 |
| Proportion(Deer) ~ Bear Density * Cougar Sex + Kittens (Any Age) + (1 \| CougarID) | 7 | -2988.699 | 61.295 |
| Proportion(Deer) ~ Bear Occupancy * Cougar Sex + Kittens (>3 months) + (1 \| CougarID) | 7 | -2985.786 | 64.208 |
| Proportion(Deer) ~ Deer Density + Cougar Sex + Kittens (>3 months) + (1 \| CougarID) | 6 | -2985.691 | 64.303 |
| Proportion(Deer) ~ Bear Density + Cougar Sex + Kittens (>3 months) + (1 \| CougarID) | 6 | -2985.610 | 64.384 |
| Proportion(Deer) ~ Bear Density + Kittens (>3 months) + Cougar Sex + (1 \| CougarID) | 6 | -2985.610 | 64.384 |
| Proportion(Deer) ~ Bear Density * Cougar Sex + Kittens (>3 months) + (1 \| CougarID) | 7 | -2984.966 | 65.028 |
| Proportion(Deer) ~ Deer Density * Cougar Sex + Kittens (>3 months) + Bear Occupancy + (1 \| CougarID) | 8 | -2983.491 | 66.503 |

**Suppl. Table 3.** The set of 21 possible beta mixed models to predict the proportion of deer in the average cougar’s diet in Nevada’s Sierra Nevada between 2009-2012 and 2015-2017. All models were fit with one random intercept, Cougar ID, to control for the variation in individuals.

| **Model Name** | **df** | **AIC** | **deltaAIC** |
| --- | --- | --- | --- |
| **Proportion(Deer) ~ Bear Density * Cougar Sex + Kittens (Any Age) + (1 \| CougarID)** | **7** | **-1721.698** | **0** |
| Proportion(Deer) ~ Bear Density * Cougar Sex + Kittens (>3 months) + (1 \| CougarID) | 7 | -1708.663 | 13.035 |
| Proportion(Deer) ~ Bear Density + Kittens (Any Age) + Cougar Sex + (1 \| CougarID) | 6 | -1703.667 | 18.031 |
| Proportion(Deer) ~ Bear Density + Kittens (Any Age) + (1 \| CougarID) | 5 | -1702.723 | 18.975 |
| Proportion(Deer) ~ Bear Density + Year + Kittens (Any Age) + (1 \| CougarID) | 6 | -1700.735 | 20.963 |
| Proportion(Deer) ~ Bear Density * Bear Present at Kill + Kittens (Any Age) + Year + (1 \| CougarID) | 8 | -1699.223 | 22.475 |
| Proportion(Deer) ~ Bear Density + Cougar Sex + Kittens (>3 months) + (1 \| CougarID) | 6 | -1687.900 | 33.798 |
| Proportion(Deer) ~ Bear Density + Cougar Sex + Kittens (>3 months) + (1 \| CougarID) | 6 | -1687.900 | 33.798 |
| Proportion(Deer) ~ Bear Density + Kittens (>3 months) + Cougar Sex + (1 \| CougarID) | 6 | -1687.900 | 33.798 |
| Proportion(Deer) ~ Bear Density + Cougar Sex + Year + (1 \| CougarID) | 6 | -1687.899 | 33.799 |
| Proportion(Deer) ~ Bear Density + Cougar Sex + Year + (1 \| CougarID) | 6 | -1687.899 | 33.799 |
| Proportion(Deer) ~ Bear Density + Cougar Sex + (1 \| CougarID) | 5 | -1687.573 | 34.125 |
| Proportion(Deer) ~ Bear Density + Year + (1 \| CougarID) | 5 | -1686.042 | 35.656 |
| Proportion(Deer) ~ Bear Density + Year + Kittens (>3 months) + (1 \| CougarID) | 6 | -1685.677 | 36.021 |
| Proportion(Deer) ~ Deer Density + Year + (1 \| CougarID) | 5 | -1658.894 | 62.804 |
| Proportion(Deer) ~ Deer Density + Cougar Sex + Kittens (Any Age) + Year + (1 \| CougarID) | 7 | -1658.682 | 63.016 |
| Proportion(Deer) ~ Deer Density + Cougar Sex + Kittens (>3 months) + (1 \| CougarID) | 6 | -1644.895 | 76.803 |
| Proportion(Deer) ~ Cougar Sex + Kittens (Any Age) + Year + (1 \| CougarID) | 6 | -1632.662 | 89.036 |
| Proportion(Deer) ~ Cougar Sex + Kittens (Any Age) + Year + (1 \| CougarID) | 6 | -1632.662 | 89.036 |
| Proportion(Deer) ~ Cougar Sex + Year + (1 \| CougarID) | 5 | -1627.133 | 94.565 |
| Proportion(Deer) ~ Cougar Sex + Kittens (>3 months) + Year + (1 \| CougarID) | 6 | -1625.612 | 96.086 |

**Suppl. Table 4.** The set of 25 possible beta mixed models to predict the proportion of deer in the average cougar’s diet in Nevada’s Great Basin between 2009-2012 and 2015-2017. All models were fit with one random intercept, Cougar ID, to control for the variation in individuals.

| **Model Name** | **df** | **AIC** | **deltaAIC** |
| --- | --- | --- | --- |
| **Proportion(Deer) ~ Bear Density + Cougar Sex + Year + (1 \| CougarID)** | **6** | **-1449.547** | **0** |
| **Proportion(Deer) ~ Bear Density + Year + (1 \| CougarID)** | **5** | **-1448.772** | **0.775** |
| **Proportion(Deer) ~ Bear Density * Cougar Sex + Year + (1 \| CougarID)** | **7** | **-1447.593** | **1.954** |
| Proportion(Deer) ~ Bear Density + Year + Kittens (>3 months) + (1 \| CougarID) | 6 | -1447.269 | 2.278 |
| Proportion(Deer) ~ Bear Density + Year + Kittens (Any Age) + (1 \| CougarID) | 6 | -1446.790 | 2.757 |
| Proportion(Deer) ~ Bear Density * Bear Present at Kill + Kittens (Any Age) + Year + (1 \| CougarID) | 8 | -1446.580 | 2.967 |
| Proportion(Deer) ~ Cougar Sex + Year + (1 \| CougarID) | 5 | -1446.347 | 3.2 |
| Proportion(Deer) ~ Cougar Sex + Kittens (>3 months) + Year + (1 \| CougarID) | 6 | -1445.252 | 4.295 |
| Proportion(Deer) ~ Deer Density + Year + (1 \| CougarID) | 5 | -1444.646 | 4.901 |
| Proportion(Deer) ~ Cougar Sex + Kittens (Any Age) + Year + (1 \| CougarID) | 6 | -1444.584 | 4.963 |
| Proportion(Deer) ~ Cougar Sex + Kittens (Any Age) + Year + (1 \| CougarID) | 6 | -1444.584 | 4.963 |
| Proportion(Deer) ~ Deer Density + Cougar Sex + Kittens (Any Age) + Year + (1 \| CougarID) | 7 | -1443.460 | 6.087 |
| Proportion(Deer) ~ Bear Occupancy * Cougar Sex + Deer Density + Year + (1 \| CougarID) | 8 | -1443.335 | 6.212 |
| Proportion(Deer) ~ Bear Occupancy * Cougar Sex + Kittens (Any Age) + Year + (1 \| CougarID) | 8 | -1442.735 | 6.812 |
| Proportion(Deer) ~ Bear Density + Cougar Sex + Kittens (>3 months) + (1 \| CougarID) | 6 | -1403.082 | 46.465 |
| Proportion(Deer) ~ Bear Density + Cougar Sex + Kittens (>3 months) + (1 \| CougarID) | 6 | -1403.082 | 46.465 |
| Proportion(Deer) ~ Bear Density + Kittens (>3 months) + Cougar Sex + (1 \| CougarID) | 6 | -1403.082 | 46.465 |
| Proportion(Deer) ~ Bear Density + Cougar Sex + (1 \| CougarID) | 5 | -1402.395 | 47.152 |
| Proportion(Deer) ~ Bear Occupancy * Cougar Sex + Kittens (>3 months) + (1 \| CougarID) | 7 | -1401.792 | 47.755 |
| Proportion(Deer) ~ Bear Density + Kittens (Any Age) + Cougar Sex + (1 \| CougarID) | 6 | -1401.696 | 47.851 |
| Proportion(Deer) ~ Bear Density + Kittens (Any Age) + (1 \| CougarID) | 5 | -1401.400 | 48.147 |
| Proportion(Deer) ~ Bear Density * Cougar Sex + Kittens (>3 months) + (1 \| CougarID) | 7 | -1401.085 | 48.462 |
| Proportion(Deer) ~ Deer Density + Cougar Sex + Kittens (>3 months) + (1 \| CougarID) | 6 | -1400.901 | 48.646 |
| Proportion(Deer) ~ Bear Density * Cougar Sex + Kittens (Any Age) + (1 \| CougarID) | 7 | -1399.699 | 49.848 |
| Proportion(Deer) ~ Deer Density * Cougar Sex + Kittens (>3 months) + Bear Occupancy + (1 \| CougarID) | 8 | -1399.547 | 50 |
|  |  |  |  |

**Suppl. Table 5.** The set of 25 possible beta mixed models to predict the proportion of horse in the average cougar’s diet in Nevada’s Great Basin between 2009-2012 and 2015-2017. All models were fit with one random intercept, Cougar ID, to control for the variation in individuals.

| **Model Name** | **df** | **AIC** | **deltaAIC** |
| --- | --- | --- | --- |
| **Proportion(Horse) ~ Cougar Sex + Kittens (Any Age) + Year + (1 \| CougarID)** | **6** | **-2996.719** | **0** |
| **Proportion(Horse) ~ Bear Density + Year + Kittens (Any Age) + (1 \| CougarID)** | **6** | **-2995.370** | **1.349** |
| **Proportion(Horse) ~ Deer Density + Cougar Sex + Kittens (Any Age) + Year + (1 \| CougarID)** | **7** | **-2995.019** | **1.7** |
| Proportion(Horse) ~ Bear Occupancy * Cougar Sex + Kittens (Any Age) + Year + (1 \| CougarID) | 8 | -2994.332 | 2.387 |
| Proportion(Horse) ~ Bear Density * Bear Present at Kill + Kittens (Any Age) + Year + (1 \| CougarID) | 8 | -2991.703 | 5.016 |
| Proportion(Horse) ~ Cougar Sex + Kittens (>3 months) + Year + (1 \| CougarID) | 6 | -2983.450 | 13.269 |
| Proportion(Horse) ~ Bear Density + Kittens (>3 months) + Cougar Sex + Year + (1 \| CougarID) | 7 | -2982.693 | 14.026 |
| Proportion(Horse) ~ Bear Density + Year + Kittens (>3 months) + (1 \| CougarID) | 6 | -2982.439 | 14.28 |
| Proportion(Horse) ~ Cougar Sex + Kittens (>3 months) + Year + Deer Density + (1 \| CougarID) | 7 | -2981.639 | 15.08 |
| Proportion(Horse) ~ Cougar Sex + Year + (1 \| CougarID) | 5 | -2969.478 | 27.241 |
| Proportion(Horse) ~ Bear Density + Cougar Sex + Year + (1 \| CougarID) | 6 | -2969.239 | 27.48 |
| Proportion(Horse) ~ Bear Density + Year + (1 \| CougarID) | 5 | -2969.111 | 27.608 |
| Proportion(Horse) ~ Bear Density * Cougar Sex + Year + (1 \| CougarID) | 7 | -2967.713 | 29.006 |
| Proportion(Horse) ~ Deer Density + Year + (1 \| CougarID) | 5 | -2967.497 | 29.222 |
| Proportion(Horse) ~ Bear Occupancy * Cougar Sex + Deer Density + Year + (1 \| CougarID) | 8 | -2966.004 | 30.715 |
| Proportion(Horse) ~ Bear Density + Kittens (Any Age) + Cougar Sex + (1 \| CougarID) | 6 | -2847.946 | 148.773 |
| Proportion(Horse) ~ Bear Density + Kittens (Any Age) + (1 \| CougarID) | 5 | -2847.731 | 148.988 |
| Proportion(Horse) ~ Bear Density * Cougar Sex + Kittens (Any Age) + (1 \| CougarID) | 7 | -2845.956 | 150.763 |
| Proportion(Horse) ~ Deer Density + Cougar Sex + Kittens (>3 months) + (1 \| CougarID) | 6 | -2830.235 | 166.484 |
| Proportion(Horse) ~ Deer Density * Cougar Sex + Kittens (>3 months) + Bear Occupancy + (1 \| CougarID) | 8 | -2830.036 | 166.683 |
| Proportion(Horse) ~ Cougar Sex + Kittens (>3 months) + (1 \| CougarID) | 5 | -2829.689 | 167.03 |
| Proportion(Horse) ~ Bear Density + Cougar Sex + Kittens (>3 months) + (1 \| CougarID) | 6 | -2827.748 | 168.971 |
| Proportion(Horse) ~ Bear Occupancy * Cougar Sex + Kittens (>3 months) + (1 \| CougarID) | 7 | -2827.698 | 169.021 |
| Proportion(Horse) ~ Bear Density * Cougar Sex + Kittens (>3 months) + (1 \| CougarID) | 7 | -2825.805 | 170.914 |
| Proportion(Horse) ~ Bear Density + Cougar Sex + (1 \| CougarID) | 5 | -2771.491 | 225.228 |
|  |  |  |  |

**Suppl. Table 6.** Beta coefficients, standard errors, and log likelihood of all coefficients in the top 3 models that predict the duration of a cougar feeding bout in Nevada’s Great Basin and Sierra Nevada between 2009-2012 and 2015-2017.

| Overall Best Models for Feeding Bout duration (Sierra Nevada and Great Basin) | |  |  |  |  |
| --- | --- | --- | --- | --- | --- |
|  | **Estimate** | **Std. Error** | **df** | **t value** | **Pr(>\|t\|)** |
| Nights Spent ~ Prey Weight Class + Bear Density + Kittens (>3 months) | | | Log Likelihood: -1145.218 | |  |
| Intercept (XS Prey) | -0.5588800 | 0.13632516 | 233.4699 | -4.099610 | 5.714781e-05 |
| Small Prey | 0.2426109 | 0.1326005 | 860.9961 | 1.829638 | 6.76E-02 |
| Medium Prey | 0.7696352 | 0.15344807 | 869.6185 | 5.015607 | 6.41E-07 |
| Large Prey | 0.8821444 | 0.12807945 | 874.0392 | 6.887478 | 1.09E-11 |
| Extra Large Prey | 0.9281674 | 0.15382531 | 749.4592 | 6.033906 | 2.51E-09 |
| Bear Density | -0.2208543 | 0.0683791 | 76.5872 | -3.229851 | 1.83E-03 |
| Kittens (>3 months) | -0.3297698 | 0.09206753 | 407.7288 | -3.581825 | 3.82E-04 |

**Suppl. Table 7.** Beta coefficients, standard errors, and log likelihood of all coefficients in the top models that predict the proportion of deer in the average cougar’s diet in Nevada’s Sierra Nevada and Great Basin between 2009-2012 and 2015-2017.

| A. Overall Best Models for Prey Composition (Sierra Nevada and Great Basin) | |  |  |  |
| --- | --- | --- | --- | --- |
|  | **Estimate** | **Std. Error** | **z value** | **Pr(>\|z\|)** |
| Percent(Deer) ~ Bear Density + Cougar Sex + Year | | | Log Likelihood: 1530.997 | |
| Intercept | 819.2190 | 101.2049 | 8.0947 | 0.0000 |
| Bear Density | 0.1697 | 0.0833 | 2.0365 | 0.0417 |
| Cougar Sex Male | -1.9381 | 0.9484 | -2.0436 | 0.0410 |
| Year | -0.4066 | 0.0503 | -8.0864 | 0.0000 |
|  |  |  |  |  |
| Percent(Deer) ~ Bear Density + Cougar Sex + Kittens (Any Age) + Year | | | Log Likelihood: 1531.941 | |
| Intercept | 803.8935 | 102.0746 | 7.8755 | 0.0000 |
| Bear Density | 0.1891 | 0.0853 | 2.2160 | 0.0267 |
| Cougar Sex Male | -1.8744 | 0.9412 | -1.9914 | 0.0464 |
| Kittens (Any Age) | 0.1523 | 0.1105 | 1.3776 | 0.1683 |
| Year | -0.3990 | 0.0507 | -7.8682 | 0.0000 |
|  |  |  |  |  |
| Percent(Deer) ~ Bear Density * Cougar Sex + Year | | | Log Likelihood: 1531.554 | |
| Intercept | 816.0445 | 100.9450 | 8.0841 | 0.0000 |
| Bear Density | 0.1219 | 0.0950 | 1.2835 | 0.1993 |
| Cougar Sex Male | -1.9200 | 0.9497 | -2.0218 | 0.0432 |
| Year | -0.4050 | 0.0502 | -8.0758 | 0.0000 |
| Bear Density:Cougar Sex Male | 0.2031 | 0.1932 | 1.0513 | 0.2931 |
|  |  |  |  |  |
| Percent(Deer) ~ Bear Density * Bear Present at Kill + Kittens (Any Age) + Year | | | Log Likelihood: 1532.517 | |
| Intercept | 809.1689 | 102.5610 | 7.8896 | 0.0000 |
| Bear Density | 0.2108 | 0.0869 | 2.4243 | 0.0153 |
| Bear Present at Kill | 0.1594 | 0.0745 | 2.1381 | 0.0325 |
| Kittens (Any Age) | 0.1569 | 0.1105 | 1.4200 | 0.1556 |
| Year | -0.4019 | 0.0510 | -7.8872 | 0.0000 |
| Bear Density:Bear Present at Kill | -0.1255 | 0.0790 | -1.5885 | 0.1122 |
|  |  |  |  |  |
| Percent(Deer) ~ Bear Density * Cougar Sex + Kittens (>3 months) + Year | | | Log Likelihood: 1532.436 | |
| Intercept | 817.0585 | 100.5717 | 8.1241 | 0.0000 |
| Bear Density | 0.1091 | 0.0948 | 1.1501 | 0.2501 |
| Cougar Sex Male | -1.9611 | 0.9537 | -2.0563 | 0.0398 |
| Kittens (>3 months) Yes | -0.1252 | 0.0945 | -1.3251 | 0.1851 |
| Year | -0.4055 | 0.0500 | -8.1154 | 0.0000 |
| Bear Density:Cougar Sex Male | 0.2158 | 0.1931 | 1.1177 | 0.2637 |
|  |  |  |  |  |
| Percent(Deer) ~ Bear Density * Cougar Sex + Kittens (Any Age) + Year | | | Log Likelihood: 1532.366 | |
| Intercept | 801.7552 | 101.8474 | 7.8721 | 0.0000 |
| Bear Density | 0.1451 | 0.0977 | 1.4842 | 0.1378 |
| Cougar Sex Male | -1.8628 | 0.9429 | -1.9757 | 0.0482 |
| Kittens (Any Age) | 0.1417 | 0.1110 | 1.2765 | 0.2018 |
| Year | -0.3979 | 0.0506 | -7.8647 | 0.0000 |
| Bear Density:Cougar Sex Male | 0.1787 | 0.1946 | 0.9181 | 0.3586 |
|  |  |  |  |  |
| Percent(Deer) ~ Deer Density + Cougar Sex + Kittens (Any Age) + Year | | | Log Likelihood: 1531.182 | |
| Intercept | 790.0792 | 101.3162 | 7.7982 | 0.0000 |
| Deer Density | 0.0852 | 0.0462 | 1.8428 | 0.0654 |
| Cougar Sex Male | -1.9032 | 0.9716 | -1.9588 | 0.0501 |
| Kittens (Any Age) | 0.1354 | 0.1096 | 1.2354 | 0.2167 |
| Year | -0.3921 | 0.0503 | -7.7908 | 0.0000 |
|  |  |  |  |  |
| Percent(Deer) ~ Bear Density + Year + Kittens (Any Age) | | | Log Likelihood: 1530.067 | |
| Intercept | 802.1839 | 102.5646 | 7.8213 | 0.0000 |
| Bear Density | 0.1868 | 0.0854 | 2.1872 | 0.0287 |
| Year | -0.3985 | 0.0510 | -7.8187 | 0.0000 |
| Kittens (Any Age) | 0.1594 | 0.1105 | 1.4425 | 0.1492 |
|  |  |  |  |  |
| Percent(Deer) ~ Bear Occupancy * Cougar Sex + Deer Density + Year | | | Log Likelihood: 1532.048 | |
| Intercept | 805.4260 | 100.0778 | 8.0480 | 0.0000 |
| Bear Occupancy Yes | 0.9132 | 0.4850 | 1.8830 | 0.0597 |
| Cougar Sex Male | -1.2982 | 0.9598 | -1.3526 | 0.1762 |
| Deer Density | 0.0681 | 0.0466 | 1.4616 | 0.1439 |
| Year | -0.4001 | 0.0497 | -8.0461 | 0.0000 |
| Bear Occupancy Yes:Cougar Sex Male | -0.9250 | 0.5095 | -1.8155 | 0.0695 |
|  |  |  |  |  |
| Percent(Deer) ~ Bear Density + Year | | | Log Likelihood: 1529.032 | |
| Intercept | 818.5303 | 101.6572 | 8.0519 | 0.0000 |
| Bear Density | 0.1663 | 0.0833 | 1.9960 | 0.0459 |
| Year | -0.4066 | 0.0505 | -8.0487 | 0.0000 |
|  |  |  |  |  |
|  |  |  |  |  |
|  |  |  |  |  |
| B. Best Sierra Nevada Model for Prey Composition | |  |  |  |
| Percent(Deer) ~ Bear Density * Cougar Sex + Kittens (Any Age) | | | Log Likelihood: 867.849 | |
| Intercept | 15.5041 | 1.2203 | 12.7048 | 0.0000 |
| Bear Density | -11.4545 | 1.0293 | -11.1281 | 0.0000 |
| Cougar Sex Male | -9.5630 | 1.8324 | -5.2188 | 0.0000 |
| Kittens (Any Age) | 0.4847 | 0.1089 | 4.4502 | 0.0000 |
| Bear Density:Cougar Sex Male | 7.1878 | 1.5073 | 4.7687 | 0.0000 |
|  |  |  |  |  |
|  |  |  |  |  |
| C. Best Great Basin Model for Prey Composition | |  |  |  |
| Percent(Deer) ~ Bear Density + Cougar Sex + Year | | | Log Likelihood: 730.7737 | |
| Intercept | 1144.6294 | 166.3071 | 6.8826 | 0.0000 |
| Bear Density | 0.3520 | 0.1557 | 2.2610 | 0.0238 |
| Cougar Sex Male | -2.2213 | 1.3000 | -1.7087 | 0.0875 |
| Year | -0.5686 | 0.0826 | -6.8833 | 0.0000 |
|  |  |  |  |  |
| Percent(Deer) ~ Bear Density + Year | | | Log Likelihood: 729.386 | |
| Intercept | 1143.8043 | 165.9892 | 6.8908 | 0.0000 |
| Bear Density | 0.3484 | 0.1555 | 2.2405 | 0.0251 |
| Year | -0.5685 | 0.0825 | -6.8939 | 0.0000 |
|  |  |  |  |  |
| Percent(Deer) ~ Bear Density * Cougar Sex + Year | | | Log Likelihood: 729.3949 | |
| (Intercept) | 1144.8535 | 166.4685 | 6.8773 | 0.0000 |
| Bear Density | 0.4017 | 0.2801 | 1.4342 | 0.1515 |
| Cougar Sex Male | -2.2670 | 1.3115 | -1.7285 | 0.0839 |
| Year | -0.5687 | 0.0827 | -6.8778 | 0.0000 |
| Bear Density:Cougar Sex Male | -0.0719 | 0.3363 | -0.2139 | 0.8306 |
|  |  |  |  |  |
| D. Best Great Basin Model for Horse Composition | |  |  |  |
| Percent(Horse) ~ Cougar Sex + Kittens (Any Age) +  Year | | | Log Likelihood: 1504.36 | |
| (Intercept) | -1888.5946 | 143.19763 | -13.188728 | 1.02E-39 |
| Cougar Sex Male | 3.7261245 | 2.37149643 | 1.571212 | 1.16E-01 |
| Kittens (Any Age) | 0.7097172 | 0.13128118 | 5.406085 | 6.44E-08 |
| Year | 0.9369354 | 0.07112831 | 13.172468 | 1.26E-39 |
|  |  |  |  |  |
| Percent(Horse) ~ Bear Density + Year +  Kittens (Any Age) | | | Log Likelihood: 1503.685 | |
| (Intercept) | -1897.5415 | 143.356074 | -13.236562 | 5.40E-40 |
| Bear Density | -0.18156 | 0.1841829 | -0.9857594 | 3.24E-01 |
| Year | 0.9419414 | 0.0712245 | 13.2249629 | 6.30E-40 |
| Kittens (Any Age) | 0.6996043 | 0.13148 | 5.3209926 | 1.03E-07 |
|  |  |  |  |  |
| Percent(Horse) ~ Deer Density + Cougar Sex +  Kittens (Any Age) + Year | | | Log Likelihood: 1504.51 | |
| (Intercept) | -1.88E+03 | 143.842802 | -13.07443 | 4.61E-39 |
| Deer Density | 3.55E-02 | 0.06453993 | 0.5501662 | 5.82E-01 |
| Cougar Sex Male | 3.72E+00 | 2.3674748 | 1.5701726 | 1.16E-01 |
| Kittens (Any Age) | 7.11E-01 | 0.13113807 | 5.4245044 | 5.81E-08 |
| Year | 9.33E-01 | 0.07144702 | 13.0586926 | 5.67E-39 |

**Supplementary Figure 1.** Estimated densities of black bears per 100 km^2^ in each study site with a resident bear population over the study time period, from 2009 to 2017. Data from NDOW (2018). The Carson Range is located within the Sierra Nevada site, and the Pine Nut and Sweetwater Ranges are located within the Great Basin site.

**
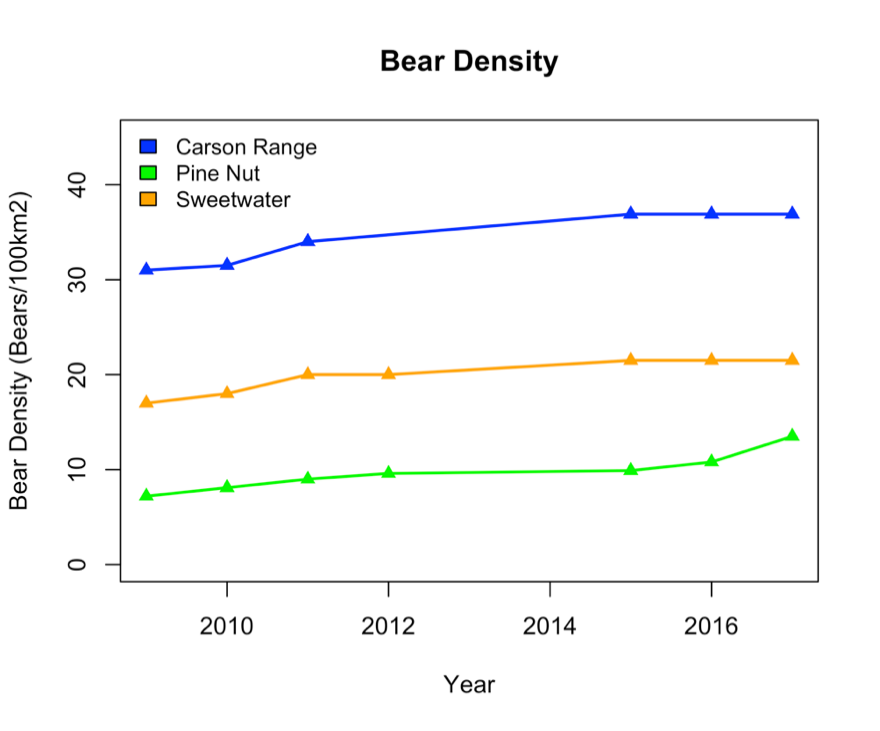
**

**Supplementary Figure 2.** Percent of cougar kills visited by black bears in each study site with a resident bear population during the months of March to October, from 2009-2012 and 2015-2017. Percentages were calculated using the proportion of all cougar kill-sites where prey items were found which also had bear scavenging evidence present at the time of the kill site investigation. The Carson Range is located within the Sierra Nevada site, and the Pine Nut and Sweetwater Ranges are located within the Great Basin site.

**
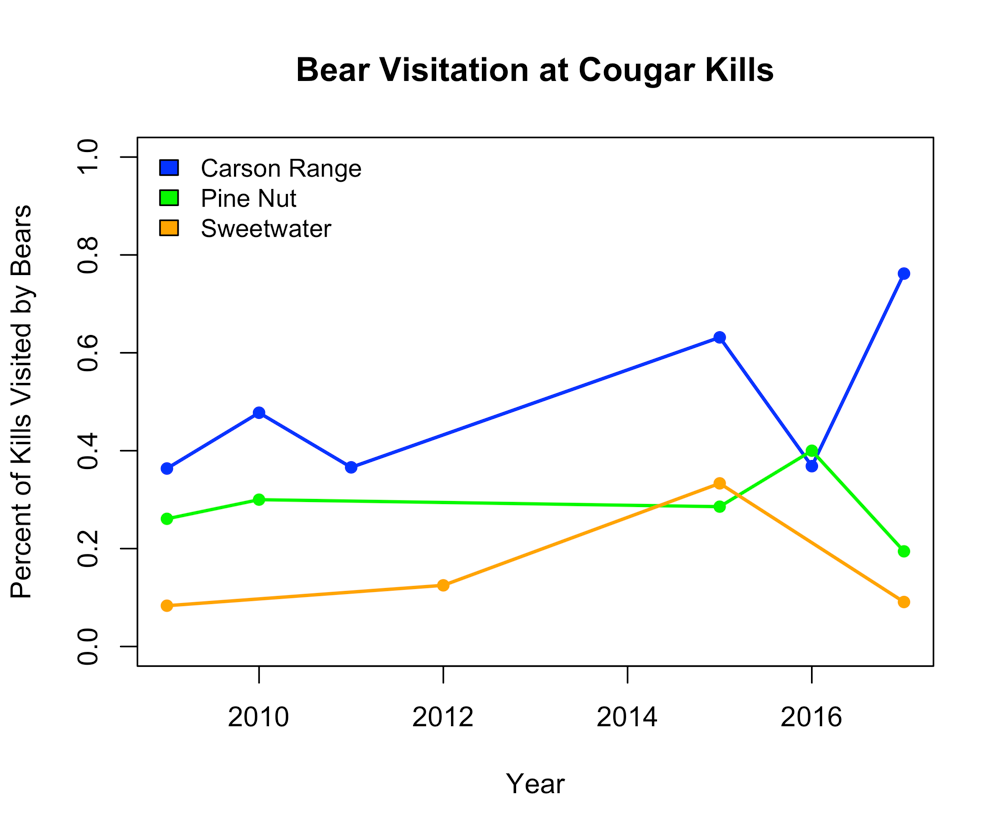
**
